# Clinical and Genetic Analysis of L-2-Hydroxyglutaric Aciduria Caused by a Novel *L2HGDH* Mutation with a Concurrent *RYR1* Variant

**DOI:** 10.3390/genes17070735

**Published:** 2026-06-26

**Authors:** Zahra Beyzaei, Seyed Mohsen Dehghani, Bita Geramizadeh, Ralf Weiskirchen

**Affiliations:** 1 Shiraz Transplant Research Center (STRC), Shiraz University of Medical Sciences, Shiraz 7193711351, Iran; z.beyzaei@gmail.com (Z.B.); geramib@gmail.com (B.G.); 2 Gastroenterology and Hepatology Research Center, Shiraz University of Medical Sciences, Shiraz 7193711351, Iran; dehghanism@sums.ac.ir; 3 Institute of Molecular Pathobiochemistry, Experimental Gene Therapy and Clinical Chemistry (IFMPEGKC) RWTH University Hospital Aachen, D-52074 Aachen, Germany

**Keywords:** L-2-hydroxyglutaric aciduria, *RYR1*, *L2HGDH*, novel mutations, exome sequencing

## Abstract

**Background/Objectives**: L-2-hydroxyglutaric aciduria (L2HGA) is a rare autosomal recessive neurometabolic disorder marked by developmental delay, intellectual disability, and progressive movement abnormalities. Variants in *RYR1* can cause congenital myopathies, but data on the co-occurrence of variants in populations are limited. The aim of this study was to characterize the clinical and genetic basis of the neurometabolic and neuromuscular abnormalities and to investigate the potential interaction between the identified variants. **Methods**: Patients with complex, previously undiagnosed clinical presentations underwent neurological evaluation, including brain magnetic resonance imaging, electromyography, biochemical testing, and whole-exome sequencing (WES). Identified variants were analyzed in silico and confirmed by Sanger sequencing in the patient and her parents. Three cases were reviewed, and one of these patients exhibited developmental delay, hypotonia, intellectual disability, and progressive motor dysfunction. Biochemical tests revealed markedly elevated urinary 2-hydroxyglutaric acid levels, consistent with L2HGA. **Results**: WES identified a homozygous likely pathogenic variant in *L2HGDH* (c.589_590insGGC, p.Q197insG), confirming the molecular diagnosis of L2HGA. In addition, a heterozygous missense variant in *RYR1* (c.7268T>A, p.M2423K), classified as a variant of uncertain significance, was detected and was inherited from her mildly affected father. The *L2HGDH* variant explains the neurometabolic phenotype of the patient, whereas the *RYR1* variant remains of uncertain significance, and its clinical contribution cannot be clearly established. **Conclusions**: To our knowledge, this case illustrates the co-occurrence of a likely pathogenic *L2HGDH* variant and a heterozygous *RYR1* variant of uncertain significance. The findings expand the mutational spectrum of L2HGA and underscore the value of comprehensive genomic testing in complex neurometabolic and neuromuscular disorders.

## 1. Introduction

L-2-hydroxyglutaric aciduria (L2HGA) is part of the group of diseases named cerebral organic acidurias, which are neurometabolic disorders characterized by the accumulation of organic acids in the brain [1]. It is characterized by psychomotor delay, macrocephaly, progressive ataxia, and impaired language [2]. L2HGA is caused by mutations in the *L2HGDH* gene, which is located on chromosome 14q22.1 and is inherited in an autosomal recessive manner. It consists of 10 exons, and its transcript has an open reading frame of 2064 bp, encoding a polypeptide of 463 amino acids. In addition, it has a shorter isoform (ENST00000261699) [3].

Although L2HGA is considered a rare disorder, more than 300 cases have been described in the literature to date, with variable clinical severity. The estimated prevalence is <1 per 1,000,000 live births, although this may be underreported due to misdiagnosis or a lack of access to genetic testing in certain regions. Higher prevalence has been observed in populations with a high rate of consanguinity, such as those in parts of the Middle East, North Africa, and South Asia, where founder effects may play a role in its distribution. Despite its rarity, early diagnosis is crucial for supportive management and genetic counseling.

“*RYR1*-related diseases” is an umbrella term. There is a wide spectrum of *RYR1*-related subtypes that impact the neuromuscular system in humans. These diseases can be inherited in an autosomal dominant or autosomal recessive manner, or they may arise from *de novo* variants. The ryanodine receptor 1 gene (*RYR1*) encodes a calcium release channel located in the sarcoplasmic reticulum of skeletal muscle [4]. It is associated with congenital myopathies and malignant hyperthermia (MH). The *RYR1* gene is one of the largest genes in the human genome and is composed of 106 exons and encodes 5038 amino acids [5]. Limited information is currently available on *RYR1* variants in populations from the Middle East.

We report a child with a complex clinical presentation in whom genetic analysis revealed a likely pathogenic variant in *L2HGDH* and an additional variant in *RYR1*.

## 2. Materials and Methods

### 2.1. Study Population and Data Collection

We conducted a retrospective, single-center analysis of pediatric patients (<18 years) who were identified in the hospital database as complex and previously undiagnosed cases. This patient had undergone neurological examination, brain magnetic resonance imaging (MRI), and electromyography (EMG), as well as biochemical testing including urinary organic acid and acylglycine profiling, between March 2000 and June 2023 at Abu-Ali Sina Hospital, Shiraz University of Medical Sciences. Clinical and biochemical data were extracted and systematically evaluated, and three patients were identified for further analysis. Genetic analysis was performed using whole-exome sequencing (WES) on an Illumina HiSeq4000 platform with 100 bp paired-end reads and an average coverage depth of 55×. Variant calling utilized the hg19 reference genome build and the Iranome database. In silico pathogenicity predictions were performed using SIFT, PolyPhen-2, and MutationTaster, and variants were interpreted according to the American College of Medical Genetics and Genomics (ACMG) guidelines [6,7]. The variant pathogenicity was further evaluated using ensemble prediction scores (CADD and REVEL), where available. Identified variants were validated by Sanger sequencing, and parental samples were tested for segregation analysis.

### 2.2. In Silico Structure Prediction

To locate the structural context in which the variant amino acid p.M2423K is located in the RYR1 protein, we performed in silico structure predictions with AlphaFold [8]. For this purpose, we used only a sequential part of the protein ranging from amino acid 2318 (Tyr) to 2507 (Asp), according to the 5038-amino acid sequence of human full-length ryanodine receptor 1 with UniProtKB/Swiss-Prot accession number P21817.3 (https://www.ncbi.nlm.nih.gov/protein/P21817.3, accessed 22 June 2026).

## 3. Results

### 3.1. Patient and Clinical Presentation

We evaluated three patients, of whom one received a definitive diagnosis and is described in this report. A 9-year-old girl was referred for evaluation of learning disability, developmental delay, intellectual disability, motor retardation, fatigue, and progressive movement abnormalities. She was the first child of consanguineous Iranian parents (second-degree cousins). The patient was born at full term following an uncomplicated pregnancy and delivery, with a birth weight of 3.15 kg and a length of 48 cm. No perinatal or postnatal complications were reported.

At 7 years of age, she developed progressive hypotonia accompanied by loss of motor control, impaired speech and emotional dysregulation. EMG findings were consistent with a myopathic process affecting both upper and lower limbs. Brain MRI demonstrated extensive bilateral subcortical white matter hyperintensities involving both cerebral hemispheres, accompanied by mild cerebral atrophy. These findings were consistent with the characteristic neuroimaging pattern observed in L2HGA. No intracranial neoplasm was detected.

### 3.2. Biochemical Findings

Urinary organic acid and acylglycine analysis showed markedly elevated excretion of 2-hydroxyglutaric acid (172 mmol/mol creatinine; normal < 10), while methylmalonic acid levels were within the normal range. These findings were consistent with a biochemical diagnosis of L-2-hydroxyglutaric aciduria (L2HGA). Enantiomer-specific analysis was not available; therefore, biochemical differentiation between the L- and D-enantiomers could not be performed. Mild elevations in liver enzymes were also detected, including alanine aminotransferase (ALT: 32 U/L) and aspartate aminotransferase (AST: 38 U/L), with values near the upper limit of normal.

### 3.3. Genetic Evaluation

WES identified a novel homozygous variant, c.589_590insGGC (p.Q197insG), in exon 5 of the *L2HGDH* gene, classified as likely pathogenic. Both parents were heterozygous carriers. According to ACMG/AMP guidelines, the variant was classified as likely pathogenic based on PM2 (absence from population databases), PP3 (multiple computational predictions supporting a deleterious effect), and PP4 (a highly specific clinical, biochemical, and neuroimaging phenotype consistent with L-2-hydroxyglutaric aciduria). Sanger sequencing confirmed the homozygous variant in the proband and heterozygous carrier status in both parents, consistent with autosomal recessive inheritance.

**Figure 1:**
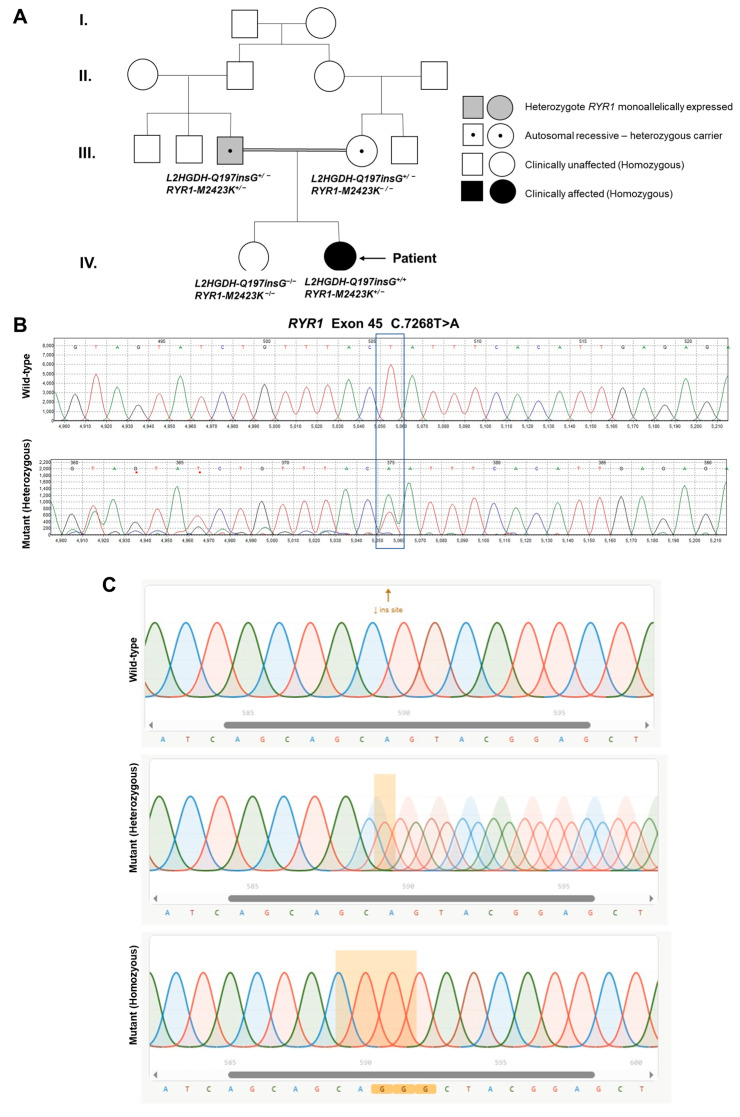
Patient family pedigree and mutation analysis. (**A**) The black arrow indicates the proband. Variants in two genes were identified: a homozygous *L2HGDH* (ENST00000261699.8) and a heterozygous RYR1 (ENST00000359596) variant inherited from the parents. *RYR1*-related disorders may follow either autosomal dominant or autosomal recessive inheritance, whereas *L2HGDH* variants cause disease through an autosomal recessive mechanism. (**B**) DNA chromatogram showing a heterozygous T-to-A transition at nucleotide 7268 of *RYR1* gene (NM_000540.3), predicting a substitution of methionine by lysine (p.Met2423Lys). (**C**) DNA chromatograms showing the c.589_590insGGC (p.Q197insG) variant in the *L2HGDH* gene. Representative traces from heterozygous and homozygous individuals are shown, demonstrating the insertion of a glycine residue (Gly) at codon 197.

Additionally, a known heterozygous missense variant, c.7268T>A (p.M2423K), in exon 45 of the *RYR1* gene was detected, classified as a variant of uncertain significance (VUS). Parental testing confirmed that the father carried the variant and exhibited muscle-related symptoms, while the mother and siblings were negative. Although this observation warrants further investigation, the currently available evidence is insufficient to establish a causal contribution of the RYR1 variant to the patient’s phenotype (Figure 1).

Exome sequencing may reveal additional variants unrelated to the primary diagnosis. In the present case, a heterozygous *RYR1* VUS was detected. Although *RYR1* variants are associated with several congenital myopathies, the heterozygous state and the current classification of this variant as a VUS preclude a definitive causal interpretation. Nevertheless, the presence of mild muscle symptoms in the father carrying the same variant raises the possibility of a phenotypic modifier or of a coincidental finding.

**Figure 2:**
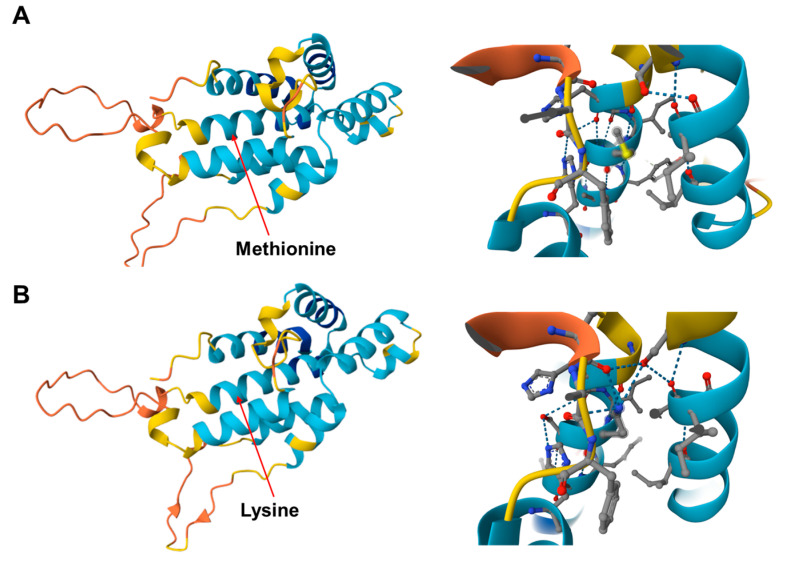
Predicted structure of the human RYR1 protein and the predicted impact of the *RYR1* gene mutation identified in this study. In this analysis, the amino acid sequence ranging from Tyr 2318 to Asp2507, according to the 5038-amino-acid sequence of the human full-length ryanodine receptor 1, was used as a template in AlphaFold structure prediction [8]. (**A**) In the depicted structure, the fold was predicted for the wild-type protein with methionine at amino acid position 2423, whereas in (**B**) the predicted fold is shown when lysine replaces methionine at this position. The right panels in (**A**,**B**) depict the proposed network of residue interactions surrounding the affected residue.

In silico analysis of the RYR1 p.Met2423Lys variant revealed a CADD score of 24. Evolutionary conservation analysis demonstrated that the affected residue is highly conserved (phyloP100 = 8.001). Structural modeling using AlphaFold was performed to assess the potential impact of the RYR1 p.Met2423Lys variant. The analysis did not predict any major alterations in the overall local protein structure. However, comparison of the wild-type and mutant models suggested changes in the network of residue interactions surrounding the affected amino acid, indicating a potential effect on local structural stability or intermolecular interactions (Figure 2).

## 4. Discussion

We report a novel homozygous variant in the *L2HGDH* gene and a known heterozygous variant in the *RYR1* gene in a 9-year-old girl born to a consanguineous Iranian family. The *L2HGDH* variant results in loss of function and was classified as likely pathogenic based on ACMG guidelines, considering criteria such as PS1, PM2, and PP3. In silico analysis also supported its deleterious effect, reinforcing its likely pathogenic classification. At present, there is no direct functional evidence of a likely pathogenic interaction between *L2HGDH* and *RYR1*. Therefore, this case should not be interpreted as a confirmed digenic disorder or a dual molecular diagnosis. Although the identified *RYR1* variant could potentially influence certain clinical features, the currently available evidence is insufficient to establish a modifying effect, and its clinical significance remains uncertain.

Concerning mutations in the *RYR1* gene, both dominant and recessive variants have been reported, with considerable phenotypic overlap between the two inheritance modes. As a general principle, dominant heterozygous variants are most commonly associated with central core disease (CCD) and malignant hyperthermia susceptibility (MHS), while recessive biallelic variants predominantly underlie multiminicore disease (MmD), centronuclear myopathy (CNM), and congenital fibre type disproportion (CFTD); however, these genotype–phenotype correlations are not absolute [9].

The *RYR1* variant c.7268T>A (p.Met2423Lys) carries a dual disease association: it was originally identified by Jungbluth et al. (2005) in three siblings with MmD and external ophthalmoplegia in a recessive context [10], where apparent heterozygosity on genomic DNA was later confirmed by Klein et al. (2012) to represent compound heterozygosity with a second allele (p.Trp661X) in trans [11]. ClinVar (accession RCV002504782; OMIM 180901.0027) also records this variant in association with autosomal dominant CCD and MHS1. In the present patient, c.7268T>A was detected in the heterozygous state without identification of a second pathogenic *RYR1* allele. Given the limited evidence available for this specific variant in heterozygous carriers, the absence of classical CCD histopathological findings, and the lack of a personal or family history of malignant hyperthermia, the variant is currently classified as a VUS. Nevertheless, because RYR1 variants may be associated with malignant hyperthermia susceptibility, appropriate anesthetic precautions should be considered in future surgical settings [12,13]. Although in silico analyses predict a possible functional effect, current evidence is insufficient to establish a definitive genotype–phenotype correlation or support a causal contribution of this variant to the clinical findings observed in this family. The variant therefore remains classified as a VUS. The father was found to carry the same heterozygous RYR1 variant and reported mild muscle-related symptoms. However, no functional studies, muscle biopsy findings, or comprehensive neuromuscular evaluation are available to support a causal association. Consequently, any relationship between the variant and the reported symptoms remains speculative. Further functional studies and additional well-characterized cases are required to clarify the clinical significance of this variant.

Molecular characterization of *L2HGDH* has been performed in more than 33 families with L2HGA to date, providing valuable insights into the genetic underpinnings of the condition. Previous studies have identified a diverse array of variants in the *L2HGDH* gene, suggesting the presence of multiple disease-related haplotypes and a potentially higher incidence of carriers than initially anticipated [2,14]. These findings highlight the complex genetic landscape of L2HGA and suggest that the condition may be more prevalent than previously recognized. Notably, the Mediterranean region appears to have a relatively high prevalence of L2HGA, consistent with earlier reports suggesting a regional genetic predisposition [15]. This observation underscores the importance of considering geographic and ethnic factors when assessing the incidence of rare metabolic disorders such as L2HGA. Furthermore, although the possibility of multiple origins for this condition remains a topic of ongoing debate, it is important to emphasize that these hypotheses are based on preliminary data and that additional genetic and epidemiological studies are necessary to confirm potential common ancestral origins. Comparing our case with previously reported cases, the clinical presentation of our patient aligns with the hallmark features of *L2HGA*, including developmental delay, hypotonia, and seizures, which have frequently been reported in similar cases [16,17]. However, the molecular findings in our case include variants that may provide additional insights into the spectrum of genetic alterations associated with *L2HGA*. This highlights the importance of ongoing molecular profiling and longitudinal studies to better understand the full clinical and genetic spectrum of the disease.

The molecular analysis confirmed variants in two different genes, *L2HGDH* and *RYR1*. While multiple potentially relevant genetic findings can occasionally be observed in a single individual, their clinical interpretation depends on the strength of evidence supporting each variant. Posey et al. [18] reported that among 7374 patients, 101 individuals (4.9%) had multiple disease loci and that blended or overlapping phenotypes may occur in some cases. In the present patient, the likely pathogenic *L2HGDH* variant provides a molecular explanation for the diagnosis of L-2-hydroxyglutaric aciduria. In contrast, the *RYR1* c.7268T>A (p.Met2423Lys) variant remains classified as a VUS, and its contribution to the patient’s phenotype is currently unclear. To date, no direct molecular or pathway-level interaction between *L2HGDH* and *RYR1* has been established. *L2HGDH* is involved in the mitochondrial metabolism of L-2-hydroxyglutarate, whereas *RYR1* encodes a skeletal muscle calcium-release channel essential for excitation-contraction coupling. Therefore, the coexistence of variants in these genes is currently considered an incidental finding, with no evidence supporting a shared pathogenic mechanism.

Our case illustrates that molecular analysis, particularly using WES, is an essential tool for diagnosing rare disorders. Early genetic diagnosis through exome sequencing offers numerous benefits, including improved time and cost efficiency, facilitation of appropriate treatment, provision of precise recurrence-risk counseling, and targeted patient screening when warranted [19].

## 5. Limitations of This Study

Despite the insights provided by this case, several limitations should be acknowledged. First, functional studies were not performed to evaluate the biological impact of the identified *L2HGDH* c.589_590insGGC variant or to clarify the potential effect of the *RYR1* c.7268T>A variant on skeletal muscle function. Second, the interpretation of the *RYR1* variant remains limited by its classification as a VUS and by the lack of additional affected family members available for extended segregation analysis. Furthermore, biochemical and molecular analyses were performed in a single patient, which restricts broader conclusions regarding genotype–phenotype correlations. Future functional investigations and the identification of additional patients with similar variant combinations may help clarify the potential contribution of *RYR1* variants as phenotypic modifiers in individuals with L2HGA.6.

## 6. Conclusions

In conclusion, we describe an Iranian patient with L-2-hydroxyglutaric aciduria caused by a novel homozygous variant in the *L2HGDH* gene. This report expands the known mutational spectrum of *L2HGDH* and further supports the utility of exome sequencing in the diagnosis of rare neurometabolic disorders. In addition, a heterozygous *RYR1* variant of uncertain significance was identified. However, the currently available clinical and genetic evidence is insufficient to establish its contribution to the patient’s phenotype. This case highlights the challenges associated with the interpretation of multiple rare variants detected through genomic testing and underscores the importance of comprehensive phenotypic evaluation, family segregation analysis, and functional studies for accurate variant interpretation. As genomic technologies become increasingly integrated into clinical practice, incidental and potentially contributory genetic findings are likely to be encountered more frequently, emphasizing the need for cautious and evidence-based interpretation.

## Data Availability

The data that support the findings of this study are available from Molecular Genetics Laboratory, but restrictions apply to the availability of these data, which were used under license for the current study, and so are not publicly available. Data are, however, available from the authors upon reasonable request and with permission of Molecular Genetics Laboratory. The datasets generated and/or analyzed during the current study are available in the ClinVar repository (https://www.ncbi.nlm.nih.gov/clinvar/variation/12989/, accessed 1 June 2026]. The accession number of the variant in ClinVar is as follows: RYR1 NM_000540.3:c.7268T>A (p.Met2423Lys): VCV000012989.30.

## Abbreviations

The following abbreviations are used in this manuscript:

ACMG	American College of Medical Genetics and Genomics
ALT	Alanine aminotransferase
AST	Aspartate aminotransferase
CCD	Central core disease
CFTD	Congenital fiber type disproportion
CNM	Centronuclear myopathy
EMG	Electromyography
L2HGA	L-2-hydroxyglutaric aciduria
*L2HGDH*	L-2-hydroxyglutarate dehydrogenase gene
MH	Malignant hyperthermia
MHS	Malignant hyperthermia susceptibility
MmD	Multiminicore disease
MRI	Magnetic resonance imaging
*RYR1*	Ryanodine receptor 1 gene
VUS	Variant of uncertain significance
WES	Whole-exome sequencing
